# MiR-155-Mediated Deregulation of GPER1 Plays an Important Role in the Gender Differences Related to Inflammatory Bowel Disease

**DOI:** 10.1155/2020/8811477

**Published:** 2020-09-15

**Authors:** Xiaojuan Shao, Jintao Li, Fumin Xu, Dongfeng Chen, Kaijun Liu

**Affiliations:** ^1^Department of Gastroenterology, Daping Hospital, Army Medical University, Chongqing 400042, China; ^2^Department of Military Biosafety, College of Basic Medical Sciences, Army Medical University, Chongqing 400042, China

## Abstract

**Aim:**

The incidence and clinical manifestations of inflammatory bowel disease (IBD) are thought to have gender differences, which suggests that the estrogen signaling pathway and intestinal flora may play key roles in the pathogenesis of IBD. In IBD, microRNA-155 (miR-155) is upregulated and regulates *G* protein coupled estrogen receptor (GPER1), which affects the intestinal flora. The objective of this study was to investigate the role of the estrogen receptors and miR-155 in the pathogenesis of IBD.

**Methods:**

From July 2018 to July 2019, in the Department of Gastroenterology at Daping Hospital, Army Military Medical University, a total of 50 patients with IBD were included in this study, and 24 healthy examinees were randomly selected as the control group. Colonoscopies were performed, and clinical characteristics and blood samples were collected from all of the subjects. The serum cytokine levels in the patients with IBD and the health donors were detected by ELISA, and the estrogen receptor level measurements for all of the participants were assessed by immunohistochemistry (IHC) and quantitative real-time PCR (qPCR). The miR-155 levels were detected by qPCR in all of the participants, and miR-155^−/−^ mice were used to investigate the mechanism of miR-155 in the pathogenesis of IBD.

**Results:**

The clinical characteristics and medications were different for the IBD patients when gender was considered. The male patients produced more proinflammatory cytokines, and while GPER1 expression was downregulated, miR-155 was upregulated in the patients with IBD. MiR-155 showed proinflammatory activity, while GPER1 showed an anti-inflammatory response during the pathogenesis of IBD. The miR-155^−/−^ mice showed improvements in weight loss, survival, rectal bleeding, colon length, and histopathological changes compared with the wild-type mice. Furthermore, the male miR-155^−/−^ mice showed increased inflammation compared to the female miR-155^−/−^ mice in the above aspects.

**Conclusion:**

This study presents evidence indicating that miR-155 plays a key role in the pathogenesis of IBD for the different genders. MiR-155 was upregulated and showed proinflammatory activity, whereas GPER1 showed an anti-inflammatory response during the pathogenesis of IBD. The results demonstrated that more proinflammatory cytokines and reduced GPER1 levels were observed in the male IBD patients. Thus, miR-155 was involved in the regulation of GPER1 and induced gender differences in IBD patients. MiR-155 may be a potential marker for IBD-targeted therapy.

## 1. Introduction

Inflammatory bowel diseases (IBD), which include ulcerative colitis (UC) and Crohn's disease (CD), result from deregulated inflammation in a genetically susceptible host [1]. Recently, the incidence of IBD has dramatically increased worldwide, especially in Asian countries [2]. However, the pathogenesis of IBD is not fully clarified. Environmental influences, a disbalance of intestinal microbes, and genetic susceptibility are all involved in the pathophysiology of IBD. The gut microbiota changes the community structure and functional capacity throughout the development of IBD. The ratio of the incidence of IBD in men and women is 1 : 1.5 [3], but females are 60% less likely to develop inflammation-associated colon cancer compared to males [4]. Therefore, it was hypothesized that sex hormones and their receptors may play a role in the pathogenesis of IBD and differences in the characteristics of IBD in males and females. Moreover, the formation of intestinal flora is influenced by the sex hormones that govern the gender differences [5].

Estrogen receptors, including the nuclear estrogen receptors (ER*α* and ER*β*) and the membrane receptor (*G* protein coupled estrogen receptor, GPER1), are involved in the induction of inflammation [6]. GPER1 has a higher affinity for estrogen and could mediate rapid signal transduction and transcriptional events [7], and the stimulation of GPER1 activates the MAPK, PI3K and NF-*κ*B-dependent signaling pathway as well as other cellular signaling pathways [8]. Although the activation of GPER1 reduces the production of inflammatory cytokines and increases the production of anti-inflammatory cytokines [9, 10], the precise role of GPER1 in the pathogenesis of IBD and its relationship with disease activity is not fully understood. Thus, the gut microbiome might be associated with the development of IBD. MicroRNA-155 is upregulated in a variety of inflammatory diseases and is thought to be a positive regulator of T-cell responses. MiR-155 is one of the most highly expressed miRNA in the serum samples of IBD patients, and probiotics improve intestinal inflammation by regulating miR-155 [11]. An miRNA database search revealed that GPER1 is a predicted target of MiR-155. Thus, our goal was to investigate the role of estrogen receptors in the pathogenesis of IBD and the effect of MiR-155 on the regulation of the estrogen receptor signaling pathway. This study investigated the potential mechanism of IBD, which contributes to the investigation of new therapeutic targets.

## 2. Materials and Methods

### 2.1. Participants and Colon Mucosa Sample Collection

A total of 74 patients were admitted to the Department of Gastroenterology at Daping Hospital from July 2018 to July 2019 and were included in this study. The age of participants ranged from 18 to 65 years old. The diagnosis of the patients with IBD was according to the criteria of Lennard-jones, a common diagnostic criterion, which includes clinical, endoscopic, histopathologic, and radiological diagnoses [12]. Patients were excluded from the study if any of the following conditions were met: minors, pregnancy, uncontrolled medical or other serious diseases, or psychiatric disease. Blood samples and colon specimens from an endoscopic biopsy were collected from 22 patients with Crohn's disease (CD) (15 men and 7 women), 28 patients with ulcerative colitis (UC) (16 men and 12 women), and 24 patients unrelated to the disease, which served as the control group (12 men and 12 women). This study was approved by the Ethics Committee of Army Medical Center, and all the participants provided written informed consent prior to the study initiation (chictr.org.cn, ChiCTR1800017211).

### 2.2. Classification of IBD and Disease Activity Scores

The classification of IBD was according to the Montreal classification. The Montreal classification for CD included age at diagnosis (A1 = <40y versus A2 = ≧40), disease behavior (B1 = nonstructuring and nonpenetrating versus B2 = structuring versus B3 penetrating), and disease location (L1 = ileal versus L2 = colonic versus L3 = ileocolonic versus L4 = upper). The Montreal classification for UC included extent (E1 = proctitis versus E2 = left-sided versus E3 = extensive) [13]. The disease severity of CD was measured according to Crohn's Disease Activity Index (CDAI) score [14]. CDAI scores of <150, 150–450 and > 450 were classified as the remission stage, mild, and moderate active stage and severe stage, respectively.

The Modified Mayo Disease Activity Index (MMDAI) was used for the clinical and research evaluation of UC [15]. Scores of ≤2, 3–10 and 11-12 were classified as clinical remission and mild, moderate, and severe activity, respectively.

### 2.3. Plasma Inflammatory Cytokine, Estradiol, and Testosterone Measurements

A total of 5 ml serum was collected without an anticoagulant from the patients with IBD and the health donors. The blood samples were centrifuged at 2750 rpm for 12 minutes at room temperature (RT). The serum was collected and stored at −80°C until use. All the hematological indexes were measured by an automatic biochemical analyzer. The levels of TNF*α*, IL-6, and IL-10 in the plasma were detected using a chemiluminescence immune detection system (Immulite1000, Siemens, Germany). The estradiol and testosterone levels were detected using an automatic chemiluminescence immunoanalyzer (DX1801, Beckman, USA). White blood cells (WBC), red blood cells (RBC), platelets (PLT), hemoglobin (Hgb), C reactive protein (CRP), and the erythrocyte sedimentation rate (ESR) were detected by an automatic biochemical analyzer (XE2100, Sysmex, Japan). Albumin levels were recorded by an automatic biochemical analyzer (DXC800, Beckman, USA).

### 2.4. Histopathology and Immunohistochemistry (IHC)

The colon tissues obtained from the colonoscopies were fixed in formalin. The tissues were sectioned (5 *μ*m) and mounted on glass slides. Hematoxylin and eosin staining was performed to evaluate colon inflammation. Paraffin-embedded sections were deparaffinized and dehydrated. Antigen retrieval was then performed in ethylenediaminetetraacetic acid buffer (EDTA; pH 9.0) for 20 minutes using a pressure cooker. The sections were incubated in an endogenous peroxidase blocker and a normal goat serum working fluid (ZSGB-Bio, Beijing, China) to block endogenous peroxidase and nonspecific responses. The sections were then incubated overnight at 4°C with primary antibodies against GPER1 (Abcam, Cambridge, UK), ER*α* (Proteintech, Wuhan, China), and ER*β* (Proteintech, Wuhan, China). The next day, the secondary antibodies (ZSGB-Bio, Beijing, China) were applied. The antibody binding was visualized by DAB staining (Dako, Glostrup, Denmark), and the slides were counterstained with Mayer's hematoxylin before applying glass coverslips [16].

### 2.5. Reverse Transcription and Quantitative Real-Time PCR

Colon samples from the mice were homogenized with an appropriate amount of Trizol reagent (Omega, Georgia, USA) followed by the repeated addition to an adsorption column and were centrifuged. The total RNA was extracted from the tissue samples. Specific targeting primers (Sangon, Shanghai, China) were added for the cDNA synthesis by reverse transcriptase. The microRNA was reverse transcribed into cDNA using the Bulge-Loop MicroRNA qRT Primer (Ribobio, Guangzhou, China), and this was used as a template for real-time qPCR with the TB green premix Ex Taq II (Takara Bio, Japan). The gene specific primers were as follows: ER*α*, forward primer 5′- TACTGCATCAGATCCAAGGGAA-3′, reverse primer 5′-CCTCGGGGTAGTTGTACAC-3′; ER*β*, forward primer 5′- GCTGAACGCCGTGACCGATG-3′, reverse primer 5′- ACGTGGGACAGGAGCATCAGG-3′; GPER1, forward primer 5′-TTCCGCGAGAAGATGACCATCC-3′, reverse primer 5′-TAGTACCGCTCGTGCAGGTTGA-3′; IL-6, forward primer 5′- AGACAGCCACTCACCTCTTCAG-3′, reverse primer 5′- TTCTGCCAGTGCCTCTTTGCTG-3′; IL-10, forward primer 5′-TCTCCGAGATGCCTTCAGCAGA-3′, reverse primer 5′- TCAGACAAGGCTTGGCAACCCA-3′; and TNF*α*, forward primer 5′- CGTGGAGCTGGCCGAGGAG-3′, reverse primer 5′- GCAGGCAGAAGAGCGTGGTG.

### 2.6. Western Blotting

An immunoenzymatic (Western blot) method was used to detect the protein levels of GPER1, AKT1, and NF-*κ*Bp65 in the colon biopsy specimens. The entire colon samples from the mice were homogenized in lysis buffer with an appropriate amount of phenylmethylsulfonyl fluoride, and the lysates were centrifuged at 11,750×g for 12 min. Equal amounts of protein from each sample were subjected to SDS-PAGE. After electrophoresis, the proteins were blotted onto polyvinylidene difluoride membranes and were incubated with the primary antibodies against GPER1 (1 : 1000), AKT1 (1 : 1000), and NF-*κ*Bp65 (1 : 1000) (Abcam, Cambridge, UK) overnight at 4°C. The membranes were then incubated in the corresponding secondary antibodies (Abcam, Cambridge, UK). A freshly prepared ECL kit (Beyotime, Beijing, China) was used to visualize the proteins on the membrane in the darkroom [9]. The film was scanned, and the AlphaEaseFC software processing system was used to analyze for optical density values.

### 2.7. Mice and Reagents

The miR-155^−/−^ mice were purchased from Jackson Laboratory and were housed under specific pathogen-free conditions. All the miR-155^−/−^ mice used in our study were homozygous. SPF C57BL/6 mice obtained from the laboratory animal center at the Army Medical University were maintained and bred under specific pathogen-free conditions. All the mice were between 8 and 10 weeks old. Dextran sulfate sodium salt (DSS) colitis was induced by adding 3% DSS (Sigma-Aldrich) to the drinking water for 5 days, followed by regular drinking water for 5 days. Severity of colitis was assessed using the disease activity index (DAI), which is based on the loss of body weight, stool consistency, and hematochezia. The animal experiments conformed to guidelines of animal usage in research issued by the Army Medical University.

### 2.8. Statistical Analysis

GraphPad Prism 7.0 (GraphPad Software, San Diego, CA, USA) software was used for the data and statistical analyses. Continuous variables are presented as the mean ± standard deviation. A *T*-test was used to compare the studied groups. Categorical variables are expressed as frequencies and percentages and were compared using *χ*2 tests. The statistical comparison using the four groups was performed using an analysis of variance (ANOVA) and a Mann–Whitney *U* test. A nominal *p* value <0.05 was considered statistically significant.

## 3. Results

The clinical characteristics of the male IBD patients were different compared to the female IBD patients.

Fifty patients (31 males and 19 females) who were diagnosed with IBD in the Department of Gastroenterology at Daping Hospital, Army Medical University, were enrolled in this study. In our study, the ratio of male to female was 1 : 0.47 for CD and was 1 : 0.74 for UC, respectively. The detailed demographic characteristics, laboratory characteristics, and treatment histories are shown in Supplementary Table 1. The female patients with UC were more likely than the male patients to develop extraintestinal manifestations (EIM) (*p*=0.05), while the male patients with CD were more likely than the female patients to develop various complications (*p*=0.021) (Supplementary Table 1). We further analyzed the complete medical history of the patients with IBD. In general, men suffering from CD were treated more frequently with biologics than females (*p*=0.047) (Supplementary Table 1). The PLT count and ESR level in the male patients with CD were lower compared to the female patients (*p*=0.049 and *p*=0.014, respectively) (Supplementary Table 1).

### 3.1. Gender Differences and Disease Classification

The clinical characteristics of the male patients with IBD were different compared to the female patients, indicating that gender differences existed. Thus, in order to investigate whether there was a significant difference in the disease severity and classification, we analyzed the characteristics between the male and female patients with IBD. There was no significant gender-specific difference in the patients with CD and UC, in terms of disease characteristics, according to the Montreal classification. In addition, no significant differences in the activity of the disease were found between the male and female patients (Supplementary Table 2).

**Figure 1:**
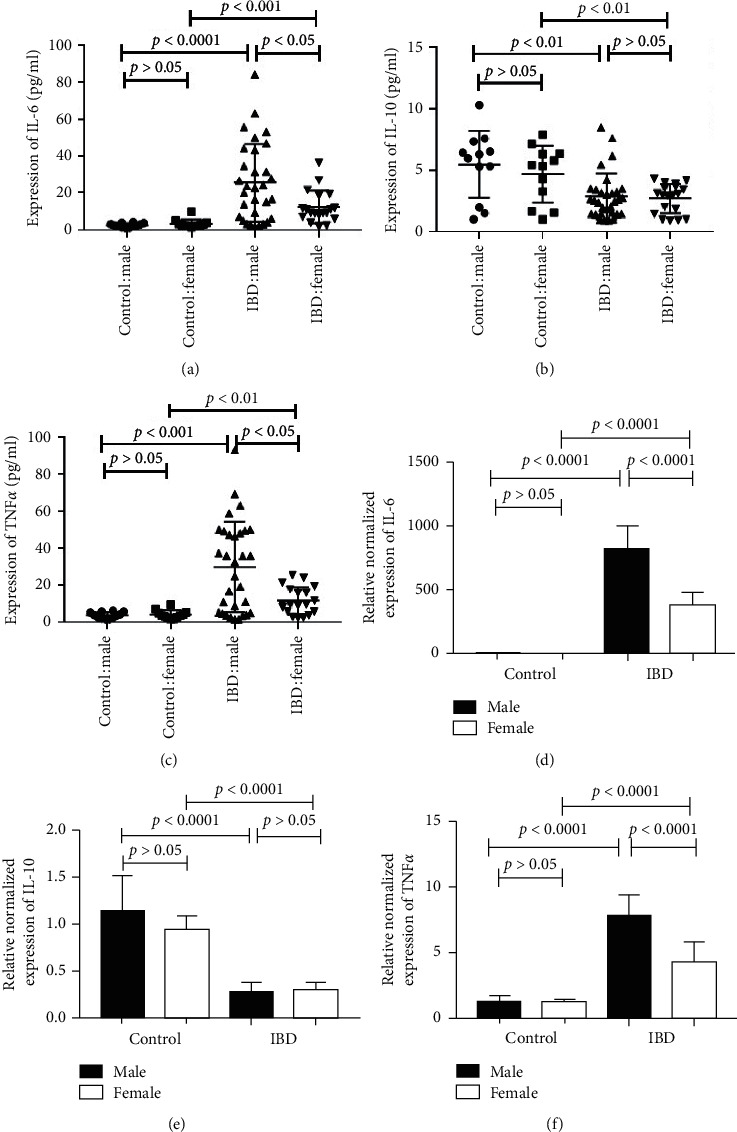
The expression of IL-6 and TNF*α* in male IBD patients is higher than in female patients. 1a and d expression of IL-6 in the serum (a) and tissue (d) of the IBD patients and control group. (b, e) Expression of IL-10 in the serum (b) and tissue (e) of the IBD patients and control group. (c, f) Expression of TNF*α* in the serum (c) and tissue (f) of the IBD patients and control. The values are presented as the mean ± SEM.

Since the clinical characteristics of the IBD patients showed a gender difference, but no significant difference was found for disease severity and classification between the male and female patients, we investigated whether there was a difference in the cytokine expression between the male and female patients. As expected, the expression of the inflammatory cytokines IL-6 and TNF*α* was higher in both the serum and tissue of the patients with IBD compared to the control group (*p* < 0.01) (Figures 1(a), 1(c), 1(d), and 1(f)), and the expression of the anti-inflammatory cytokine IL-10 was significantly lower in both the serum and tissue compared to the control group (*p* < 0.01) (Figures 1(b) and 1(e)). In addition, the expression of IL-6 and TNF*α* in the male patients with IBD was significantly higher compared to the female patients with IBD (*p* < 0.05) (Figures 1(a), 1(c), 1(d), 1(f)).

### 3.2. GPER1 Is Associated with Gender Differences in IBD Patients

The clinical characteristics and the expression of TNF*α* and IL-6 showed significant differences between male and female IBD patients, indicating that the pathogenesis of IBD may involve sex hormones and estrogen receptors. Therefore, we evaluated the sex hormone levels in the IBD patients and the control group. The levels of both estradiol and testosterone in the IBD patients were lower than in the control group (*p* < 0.05) (Supplementary Figure 1A and 1B).

**Figure 2:**
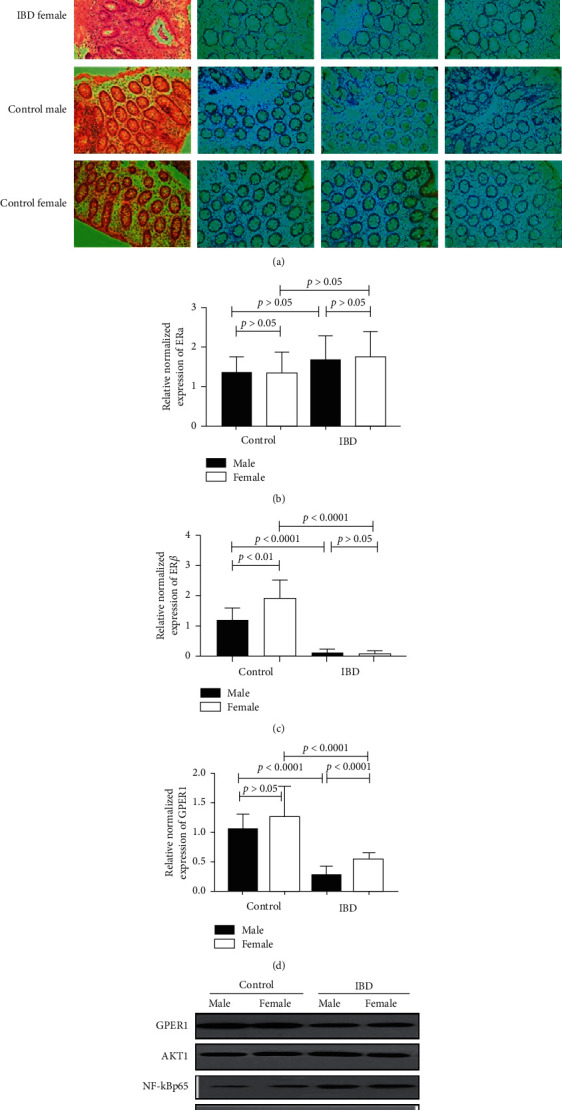
GPER1 is associated with gender differences in IBD patients. (a) Representative immunohistochemical (IHC) staining of the estrogen receptor protein in tissue (400×). (b) The mRNA expression of estrogen receptor ER*α*, ER*β* (c), and GPER1 (d) in tissue. (e) The expression of GPER1, AKT1, and NF-*κ*Bp65 is assessed by a western blot. The values are presented as the mean ± SEM.

Given that the sex hormones were lower in the IBD patients, we next assessed the expression of the estrogen receptors, that is, ER*α*, ER*β,* and GPER1, in the control and IBD groups. Compared to the control group, ER*α* expression in the IBD patients was not significantly different (*p* > 0.05), while ER*β* expression was lower in the IBD patients compared with the control group (*p* < 0.0001) (Figures 2(a)–2(c)). However, there was no significant difference between the ER*α* and ER*β* expression between the male and female IBD patients (*p* > 0.05) (Figures 2(a)–2(c)). In contrast, GPER1 expression was lower in the IBD patients compared to the control group (*p* < 0.0001). Moreover, its expression in the female patients was significantly higher compared to the male patients (*p* < 0.0001), indicating that GPER1 may be correlated with the gender differences observed in IBD (Figures 2(a) and 2(d)).

We further detected the signaling pathways downstream of GPER1. The expression of AKT1 and NF-*κ*Bp65 was significantly higher in the IBD patients than in the control group (Figure 2(e)). Furthermore, GPER1 was significantly decreased in male patients compared with female patients, while the expression of AKT1 and NF-*κ*Bp65 was higher in the male patients than in the female patients (Figure 2(e)).

### 3.3. MiR-155 Is Involved in the Mechanism Related to the Gender Differences between Male and Female IBD Patients

**Figure 3:**
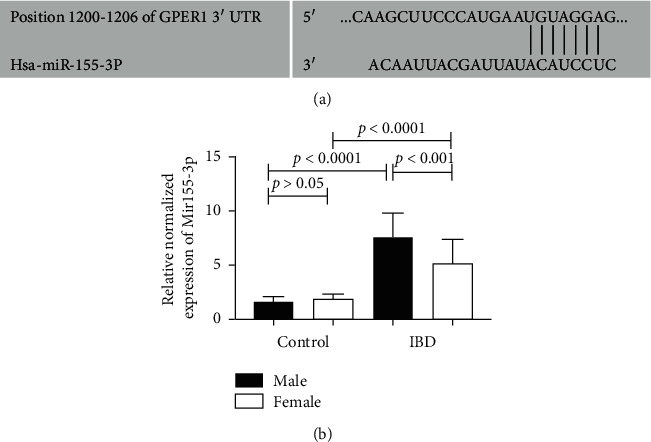
MiR-155 is involved in the regulation of GPER1. (a) GPER1 is a target of miR-155. (b) The expression of miR-155 in the colon tissues of IBD patients and the control group. The values are presented as the mean ± SEM.

The expression of GPER1 was different between the male and female IBD patients, suggesting that GPER1 might be involved in the mechanism of the gender differences observed in IBD patients. MicroRNAs play very important roles in the regulation of protein function. Using a microRNA database search, we found that the 3′UTR of GPER1 contained a potential binding site for miR-155 (Figure 3(a)). Therefore, GPER1 was a predicted target of miR-155. Here, we also found that miR-155 expression was higher in patients with IBD compared to the control group (*p* < 0.0001), and miR-155 expression was also higher in the male IBD patients compared to the female patients (*p* < 0.001) (Figure 3(b)).

### 3.4. Knock-Out of MiR-155 Could Attenuate the Intestinal Inflammation in 3% DSS-Induced Colitis

**Figure 4:**
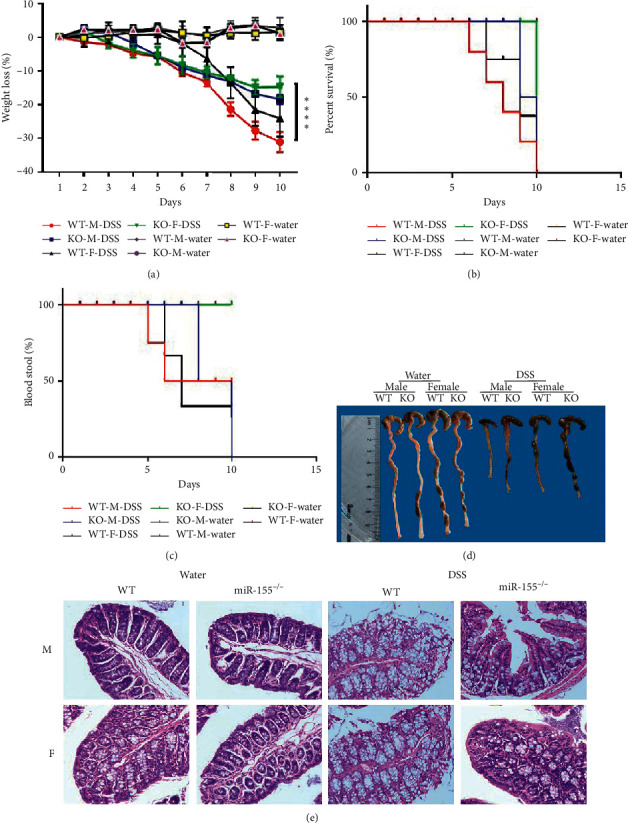
Knock-out of MiR-155 could attenuate the intestinal inflammation in 3% DSS-induced colitis. (a) Weight change, (b) Kaplan–Meier survival rate plot, (c) bloody stool, (d) representative gross colon appearance and colon length, and (e) representative H&E-stained colon cross sections (original magnification, 200×).

MiR-155 was involved in the regulation of GPER1 and may induce the gender differences observed in patients with IBD. Thus, miR-155^−/−^ mice were used to investigate the manifestations of colitis between the male and female mice. We observed that male and female miR-155^−/−^ mice showed decreased inflammation compared to the WT mice in many aspects, including weight loss (Figure 4(a)), survival (Figure 4(b)), rectal bleeding (Figure 4(c)), colon length (Figure 4(d)), and histopathological changes (Figure 4(e)). However, the male miR-155^−/−^ mice showed increased inflammation compared to the female miR-155^−/−^ mice in the above aspects (Figures 4(a)–4(e)).

## 4. Discussion

**Figure 5:**
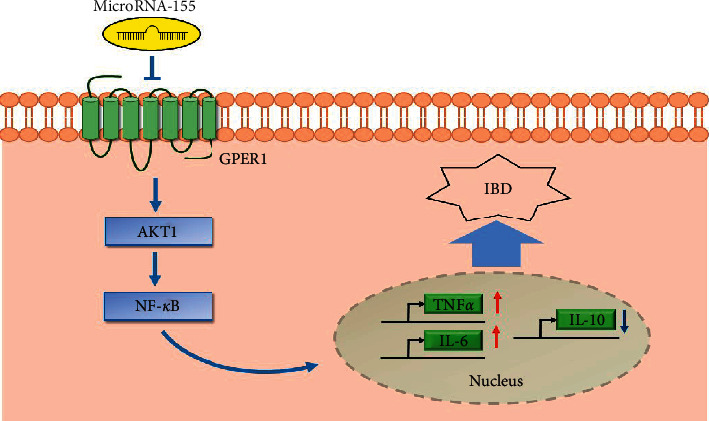
MiR-155 plays a proinflammatory role, and GPER1 is anti-inflammatory during the pathogenesis of IBD. In IBD patients, the proinflammatory cytokines IL-6 and TNF*α* were significantly higher, and the anti-inflammatory cytokine IL-10 was significantly lower.

A significant gender difference in the incidence of IBD has been reported [17]. Here, we demonstrated that male patients produce more TNF*α*, and, thus, we investigated the underlying mechanism of the gender differences observed in patients with IBD. GPER1 and miR-155 played important roles in the gender differences associated with IBD. MiR-155 played a proinflammatory role, while GPER1 was anti-inflammatory during the pathogenesis of IBD (Figure 5).

An imbalance of proinflammatory cytokines and anti-inflammatory cytokines leads to intestinal immune function and gut microbiome disorders, which is closely related to the pathogenesis of IBD. By analyzing both serum and tissue, we found that the levels of the proinflammatory cytokines IL-6 and TNF*α* were significantly higher in the patients with IBD compared to the control group, while the level of the anti-inflammatory cytokine IL-10 was lower than the control group. These findings were consistent with previous reports [18]. A disruption of the Th17/Treg balance by IL-6 is believed to be an important factor in the development of IBD [19]. Here, we found that the quantitative level of TNF*α* in the male patients was significantly higher than in the female patients. TNF*α* increases intestinal permeability in IBD by increasing the expression of epithelial myosin light chain kinase (MLCK) [20]. TNF also plays a role in the composition of the gut microbiome during development and affects the development of the immune system [21]. Anti-TNF-therapy regulates the gut microbiota and intestinal barrier function, which transforms the diversity of the gut microbiota in IBD patients toward the healthy population [22, 23]. Blocking TNF*α* is a successful targeted therapy for IBD. There were significant gender differences in TNF*α* expression, and it has been suggested that sex hormones may be related to the incidence of IBD.

More and more evidence reveals that E2 has a protective effect on inflammation-related diseases. Decreasing inflammation in mice is achieved by increasing the expression of E2 in mice, which stimulates the expression of antioxidant enzymes [24]. The concentration of estrogen metabolites (EM) appears to be closely related to the diversity of the gut microbes. For example, 16-alpha-hydroxylation of estradiol is the metabolite most closely associated with gut microbes [25]. However, there is no relevant research related to this in IBD. In contrast, estrogen improves the barrier function in intestinal epithelial cells and mucosal healing by upregulating tight junction proteins and reduces the production of inflammatory cytokines in IBD [26]. Recently, the combination of estrogen and probiotics is suggested to improve gut leakage and repair intestinal barrier function [27]. One mechanism of the protective effect of estrogen may be realized by the estrogen receptor, which is involved in the pathogenesis of IBD [4]. Our study demonstrated that, in male IBD patients, the level of E2 was significantly higher than in the control male participants. In order to further illustrate the role of the estrogen receptor, mediated by estrogen signal, in IBD, we tested the level of GPER1, ER*α,* and ER*β* in the colon. There were no significant gender differences with respect to the expression of ER*α* and ER*β* between the male and female IBD patients. However, GPER1 expression was significantly lower in the IBD patients compared to the control group, especially in the male patients with IBD. GPER1 protein was expressed in human endothelial cells, monocytes, and macrophages [10, 28]. Our immunohistochemical analysis revealed that GPER1 was localized in the cytoplasm of the intestinal epithelial cells and goblet membranes, which suggested that the expression of GPER1 was closely related to the pathogenesis of IBD. Furthermore, our data demonstrated that AKT1 and NF-*κ*Bp65, which are downstream of the PI3K-Akt signaling pathway, were significantly upregulated in IBD patients. However, there was no significant difference between males and females. In IBD, the activation of GPER1 blocks the pathway dependent on proinflammatory cytokines, and the activation of GPER1 is thought to play a key role in intestinal inflammation [10]. However, recent evidence suggests that there are significant differences in the bacterial species richness between GPER1^−/−^ and GPER1^+/+^ rats. Compared with WT GPER1^+/+^ rats, Gper1^−/−^ rats exhibit significantly reduced levels of Clostridiales under the phylum Firmicutes. Thus, the deletion of GPER1 significantly alters gut microbes [29]. MicroRNAs also modulate gut-associated metabolism by regulating gut microbes. MiR155 is highly expressed in IBD, and it might share common molecular pathways with gut microbiota, such as *Ruminococcus* [30]. Thus far, no study has shown that miR-155 has gender differences affecting gut microbes, and this will be the next goal of our research group.

In order to further study the relationship between GPER1 and microRNAs, we predicted that GPER1 is a target of miR-155 based on a miRNA database. The 3′ UTR of GPER1 contains a potential binding site for miR-155. We further detected the level of miR-155 in tissue and demonstrated that the expression of miR-155 in IBD patients was significantly increased, especially in the male IBD patients, compared to the controls. In order to verify the existence of gender differences using animal experiments, we employed miR-155^−/−^ mice and a DSS model of colitis. We found that the clinical and pathological scores of the miR-155^−/−^ mice decreased, while those of male rats increased. These data suggested that the miR-155 deficiency reduced the intestinal inflammation of DSS mice. Furthermore, we demonstrated that the female miR155^−/−^ mice were more effective at preventing inflammation.

MiR-155 is a multifunctional miRNA that is closely associated with inflammation. In particular, it is closely related to IBD and participates in the molecular changes linked to important targets and signaling pathways associated to this disease [31]. MiR-155 promotes macrophages to polarize into the M1 phenotype, which increases expression of M1 macrophages and decreases expression of M2 macrophages, thereby activating a proinflammatory pathway [32]. Probiotics improve inflammation in the colon by regulating the expression of miR-155, which is a marker involved in the colon immune response [33]. Some studies show that the clinical scores of miR-155^−/−^ mice in the acute experimental colitis model group are lower than those in the wild-type control group, reversing the pathogenesis related to colitis and reducing the systemic and inflammatory cytokines [11]. Our data confirmed that the regulation of miR-155 in IBD patients of different genders was different, which might be due to the regulation of GPER1-induced inflammation. In some chronic inflammatory diseases, such as osteoporosis, atherosclerosis, and chronic inflammatory diseases, sex dimorphic characteristics are attributed to the role of the estrogen receptor. Previously, it was reported that the expression pattern of the estrogen receptor promotes sex dimorphism, which might protect women, but not men, from inflammation [33].

Gender differences in the gut microbiota have been reported in humans. The intestinal flora of men is dominated by *Bacteroidetes* and *Prevotella*, suggesting that the difference in the gene expression of the sex chromosomes or gonadal hormone levels might affect the regulation of the gut microbiota [28]. The intestinal flora of women shows less proinflammatory functions, and, thus, females are less prone to inflammation [34]. Altogether, it is clear that the gender differences of the gut microbiota can influence the severity of IBD. Rodríguez-Nogales et al. showed that the administration of some probiotics, such as *Escherichia coli* Nissle 1917, *Lactobacillus fermentum,* and *Lactobacillus salivarius,* could attenuate the development of DSS-induced colitis by downregulating mir-155 expression [35, 36].

In conclusion, our study explored the prominent position of miR-155 in regulating GPER1 in patients with IBD of different genders. MiR-155 was a negative regulator of GPER1 in both genders. A deficiency of miR-155 led to a significant inhibition in inflammation levels for females compared with the males. Our research also had some limitations. Firstly, the underlying mechanism needs to be further clarified. Secondly, the population investigated in this study was relatively limited and, thus, needs to be further determined using a larger population. Thirdly, the gender differences related to miR-155-regulated intestinal flora need to be further confirmed. Finally, previous studies were mainly based on European and American populations, while this study mainly focused on Asian populations. In Asian populations, men are more likely to develop IBD than women [37]. More studies are needed in order to fully comprehend the mechanism of miR155 in IBD, but early results suggest that miR-155 might be a premarker for targeted therapy in patients with IBD, which warrants further studies.

## Data Availability

The data used to support the findings of this study are available from the corresponding author upon request.

## Supplementary Materials

Supplementary Materials Supplementary Table 1: demographic difference and laboratory finding between female and male IBD patient. Supplementary Table 2: classification of patients. Supplementary Figure 1: sex hormones of IBD patients decreased in IBD patients. Serum estradiol (A) and androgen (B) levels in serum. Values were shown as mean ± SEM.
