# Supplementary material for: MiR-155-Mediated Deregulation of GPER1 Plays an Important Role in the Gender Differences Related to Inflammatory Bowel Disease
Source: Can J Infect Dis Med Microbiol. 2020 Sep 15;2020:8811477. doi: 10.1155/2020/8811477 (PMC7516711; doi:10.1155/2020/8811477)
Supplement: Supplementary Materials — Supplementary Table 1: demographic difference and laboratory finding between female and male IBD patient. Supplementary Table 2: classification of patients. Supplementary Figure 1: sex hormones of IBD patients decreased in IBD patients. Serum estradiol (A) and androgen (B) levels in serum. Values were shown as mean ± SEM. [file 8811477.f1.zip › 8811477.f1/supplementary Fig1.pdf]

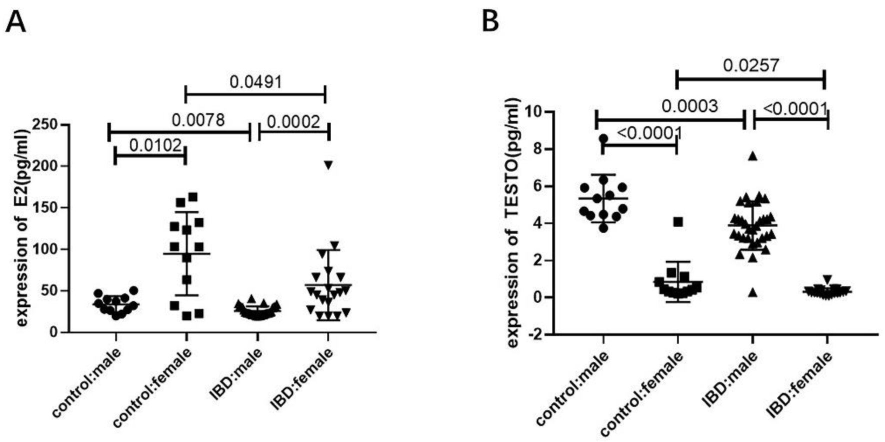

Supplementary Figure 1. Sex hormones of IBD patients decreased in IBD patients. Serum estradiol (A) and androgen (B) levels in serum. Values were shown as means ± SEM;
