# Supplementary material for: MiR-155-Mediated Deregulation of GPER1 Plays an Important Role in the Gender Differences Related to Inflammatory Bowel Disease
Source: Can J Infect Dis Med Microbiol. 2020 Sep 15;2020:8811477. doi: 10.1155/2020/8811477 (PMC7516711; doi:10.1155/2020/8811477)
Supplement: Supplementary Materials — Supplementary Table 1: demographic difference and laboratory finding between female and male IBD patient. Supplementary Table 2: classification of patients. Supplementary Figure 1: sex hormones of IBD patients decreased in IBD patients. Serum estradiol (A) and androgen (B) levels in serum. Values were shown as mean ± SEM. [file 8811477.f1.zip › 8811477.f1/supplementary table 1.pdf]

Supplementary Table 1. Demographic difference and laboratory finding between female and male IBD patient.

	CD (n=22)			UC (n=28)			Control group (n=24)		
	Male	Female	P	Male	Female	P	male	female	p
Patients, n (%)	15(68.2)	7(31.8)		16(57.1)	12(42.9)		12(50)	12(50)	
Age, years (y)	26.2±8.9	26.4±13.3	0.96	41.6±13.1	42.7±12.8	0.82	41.7±14.2	39±13.1	0.64
Duration of disease (m)	30.3±29	50.6±42.4	0.20	40.1±60.2	16.3±22.1	0.21	NA	NA	NA
Complications (%)	14(63.6)	3(13.6)	0.021	1(3.6)	3(10.7)	0.285	NA	NA	NA
EIM (%)	2(9)	3(13.6)	0.274	3(10.7)	7(25)	0.05	NA	NA	NA
5-ASA/SASP (%)	10(45.5)	5(22.7)	0.823	12(42.9)	7(25)	0.432	NA	NA	NA
Corticosteroid (%)	5(22.7)	2(9)	0.823	3(10.7)	1(3.6)	0.613	NA	NA	NA
Immunosuppressants (%)	3(13.6)	1(4.5)	0.746	1(3.6)	0(0)	1	NA	NA	NA
Biologics (%)	11(50)	2(9)	0.047	3(10.7)	0(0)	0.238	NA	NA	NA
RBC (10^{12}/L)	4.6±1.2	3.8±0.6	0.12	4.72±1.2	4.14±0.77	0.16	5.29±0.48	4.52±0.31	0.0001
WBC (10^{9}/L)	7.7±2.9	7.8±2.2	0.89	8.4±3.6	7.5±2.2	0.46	6.18±1.01	5.47±1.27	0.143
PLT (10^{9}/L)	282.4±130.7	400±101	0.049	270.3±148.8	310.7±105.5	0.431	180.7±28.6	173.3±38.3	0.601
Hgb (g/L)	128.3±31.1	91.6±20.6	0.01	122.3±29.5	112.1±26.5	0.358	144.7±11.7	128.8±11.3	0.003
ALB (g/L)	39.7±9	38.3±7	0.72	39.6±5.8	38.5±6.9	0.664	46.84±3.65	46.2±3.03	0.657
CRP (mg/L)	17.5±22	31.4±25.9	0.21	34.4±55.6	29±39.9	0.77	0.24±0.09	0.28±0.07	0.308
ESR (mm/h)	24±21.2	54.7±32.3	0.014	21.1±19.5	36.1±25	0.09	5.66±3.14	7.91±3.03	0.088

Abbreviations: EIM: extraintestinal manifestation; RBC: red blood cell; WBC: white blood cell; PLT: platelet; Hgb: hemoglobin; ALB: Albumin; CRP: C-reactive protein; ESR: erythrocyte sedimentation rate
