# Supplementary material for: MiR-155-Mediated Deregulation of GPER1 Plays an Important Role in the Gender Differences Related to Inflammatory Bowel Disease
Source: Can J Infect Dis Med Microbiol. 2020 Sep 15;2020:8811477. doi: 10.1155/2020/8811477 (PMC7516711; doi:10.1155/2020/8811477)
Supplement: Supplementary Materials — Supplementary Table 1: demographic difference and laboratory finding between female and male IBD patient. Supplementary Table 2: classification of patients. Supplementary Figure 1: sex hormones of IBD patients decreased in IBD patients. Serum estradiol (A) and androgen (B) levels in serum. Values were shown as mean ± SEM. [file 8811477.f1.zip › 8811477.f1/supplementary table 2.pdf]

Supplementary Table 2. claassification of patients

Extent of disease	Male (n=31)	female (n=19)	p
Age at presentation			
A1(<40)	19	11	0.3859
A2(≧40)	12	8	0.6301
location			
L1	3	1	0.87
L2	3	1	0.87
L3	9	4	0.33
L4	0	0	>0.9999
Behaviour			
B1	10	4	0.3891
B2	2	1	0.9828
B3	3	1	0.8952
Montreal classification			
E1	4	2	0.9259
E2	6	8	0.9259
E3	6	2	0.6864
CDAI			
<150	8	1	0.6812
150-450	5	8	0.9485
>450	2	3	0.9977
MAYO			
≤2	2	1	0.9802
3-10	10	4	0.3638
11-12	4	1	0.7323
